# A Case of Necrotizing Soft Tissue Infection Secondary to Perforated Colon Cancer

**DOI:** 10.7759/cureus.17663

**Published:** 2021-09-02

**Authors:** Nicholas Bahl, Ashley S Long, Adithi Vemuri, Tiffany Jessee

**Affiliations:** 1 Surgery, Regional Medical Center Bayonet Point, Hudson, USA; 2 Medicine, Dr. Kiran C. Patel College of Osteopathic Medicine, Nova Southeastern University, Fort Lauderdale, USA; 3 Surgery, Largo Medical Center, Largo, USA

**Keywords:** necrotizing fasciitis, necrosis, colon cancer, malignancy, soft tissue infection, necrotizing soft tissue infection, nf, nsti, cellulitis, myositis

## Abstract

Necrotizing soft tissue infections are aggressive infections that cause necrosis of muscle, fascia, and tissue. They typically follow fascial planes that lack insufficient blood supply. Early drainage and debridement are essential for survival in these patients. This is a case of a patient who presented in diabetic ketoacidosis with a necrotizing soft tissue infection localized to the left flank and abdomen with underlying colon cancer pathology.

The patient was a 54-year-old female who initially presented with acute dyspnea and left flank pain for two weeks. On admission, she was afebrile, tachycardic, tachypneic, and hypertensive. After being transferred to the ICU for diabetic ketoacidosis management, she began complaining of left abdominal pain and the CT showed concerns for a possible necrotizing soft tissue infection in the left flank region. She was taken to the operating room immediately for debridement and started on broad-spectrum antibiotics. The next day, an exploratory laparotomy was performed with a hemicolectomy and creation of an end colostomy due to concern for a perforated colonic malignancy. A final debridement was completed and a wound vacuum-assisted closure (VAC) was placed. Final pathology demonstrated well-differentiated colonic adenocarcinoma invading into the muscularis propria.

Overall, necrotizing soft tissue infections can be related to a perforated viscus especially a colonic malignancy and this case demonstrates the importance of proper surgical management and high clinical suspicion for possible underlying pathology in a soft tissue infection.

## Introduction

Necrotizing fasciitis (NF) is a subset of aggressive necrotizing skin and soft tissue infections (NSTI) that cause necrosis of the muscle, fascia, and subcutaneous tissue [1]. NF affects about 0.4 in every 100,000 people per year in the United States [1]. The infections follow certain fascial planes, which lack sufficient blood supply and leave overlying tissues initially unaffected [1]. This can delay initial diagnoses as well as surgical intervention [1]. Predisposing risk factors for NSTI include diabetes mellitus, malignancy, alcohol abuse, and chronic liver, and kidney diseases [2]. However, in over 20% of cases, the etiology is idiopathic [3-5]. 

In order to quickly and accurately diagnose NSTI, the index of suspicion must be high. Signs and symptoms can include fever, tachycardia, hypotension, shock, swelling, erythema, pain disproportionate to appearance, skin discoloration, crepitus, and subcutaneous air gas [6]. Lab values including C-reactive protein, WBC count, hemoglobin, sodium, creatinine, glucose levels can all be helpful in establishing the diagnosis [6]. Imaging studies that are useful include plain radiography, ultrasound, CT, and MRI. Management of NF includes broad-spectrum antibiotics, and importantly, early and aggressive drainage and debridement, which are the mainstay of treatment [7]. Postoperative management of the surgical wound and proper nutrition are also critical for the patient’s survival [7]. 

We present a case of a female patient that initially presented with diabetic ketoacidosis and concurrent flank pain. Upon stabilization of the diabetic ketoacidosis, she was promptly diagnosed and treated for an underlying necrotizing soft tissue infection of the left hemi abdomen and left flank which later led to the discovery of a colonic malignancy. The aim of this unique case report is to exemplify how the rapid diagnosis and treatment for a life-threatening condition can lead to optimal outcomes, as well as the benefit of remaining vigilant for possible causes of necrotizing soft tissue infection. 

## Case presentation

**Figure 1:**
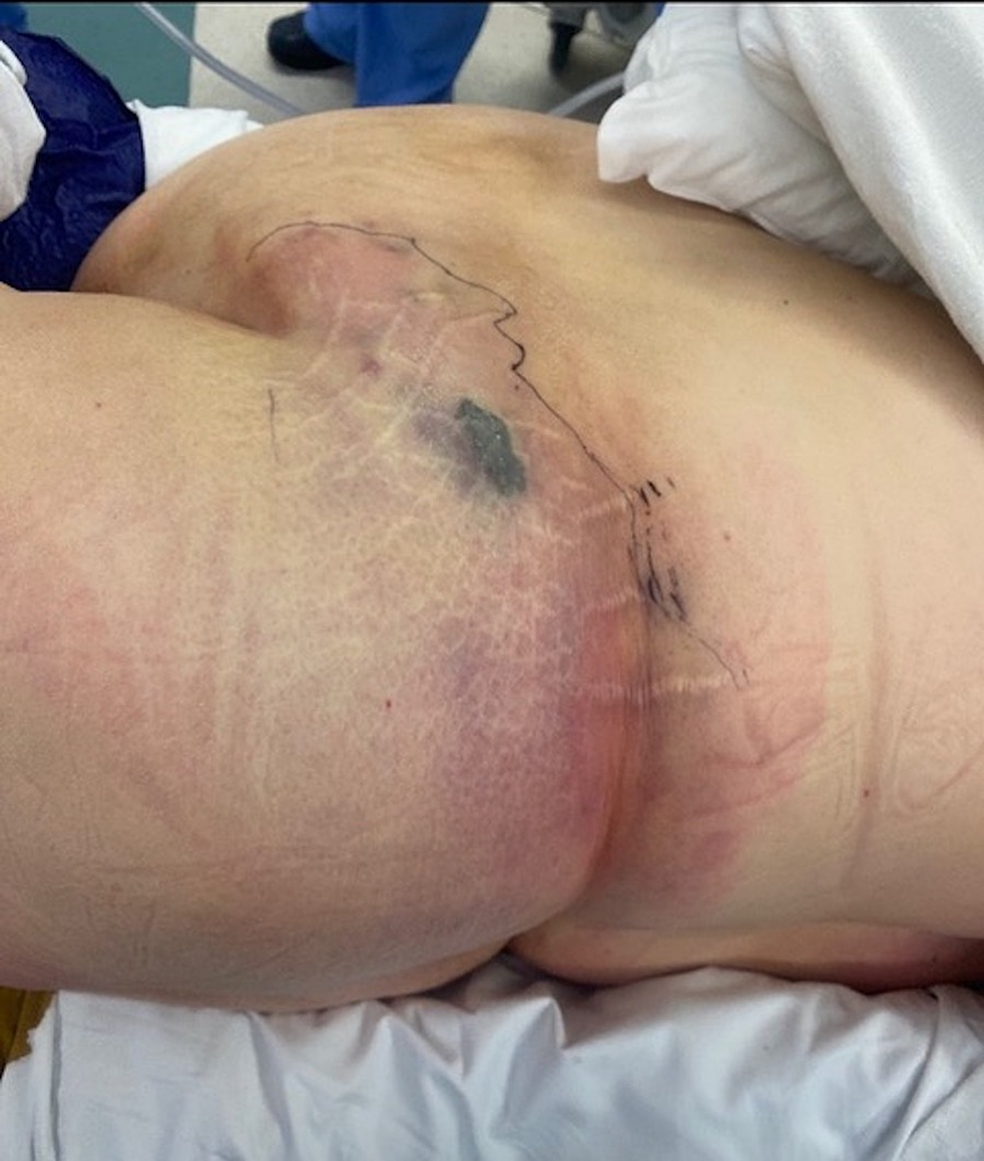
Left flank and left lower quadrant on admission

Our patient was an obese 54-year-old female who presented to the emergency department with a chief complaint of shortness of breath. She presented with a blood pressure of 176/80 mmHg, a pulse of 115 bpm, and a respiratory rate of 40 bpm. Labs on initial presentation showed a blood glucose of 653 mg/dL, leukocytosis of 17.5 K/μL, and an elevated lactic acid of 2.5 mmol/L. She had no previous diagnosis of diabetes mellitus. She was transferred to the ICU for management of diabetic ketoacidosis. After the patient was stabilized in the ICU, she started complaining of left abdominal pain. Physical exam showed a poorly defined erythema with underlying crepitus and a central stellate-shaped violaceous patch on the left flank and abdomen that was concerning for impending necrosis as shown in Figure 1.

**Figure 2:**
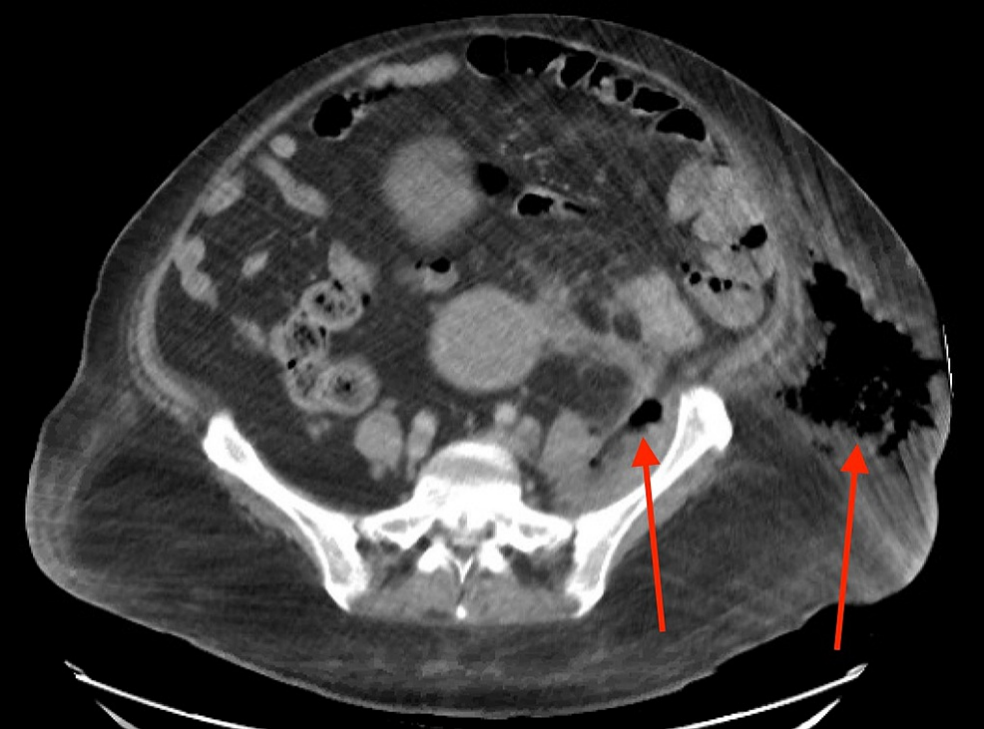
CT abdomen and pelvis showing subcutaneous air in the retroperitoneum extending into the left flank

As shown in Figure 2, the CT of the abdomen and pelvis showed a large amount of subcutaneous emphysema within the left flank soft tissues that wrapped anteriorly along the left lower lateral abdominal wall into the anterior subcutaneous soft tissues of the left lower quadrant. There was also a small tract of air extending from the left retroperitoneum posterior to the left kidney and extending along the left iliopsoas muscle inferiorly. Mild soft tissue stranding and thickening adjacent to the proximal sigmoid colon in the region of the subcutaneous air was present as well.

**Figure 3:**
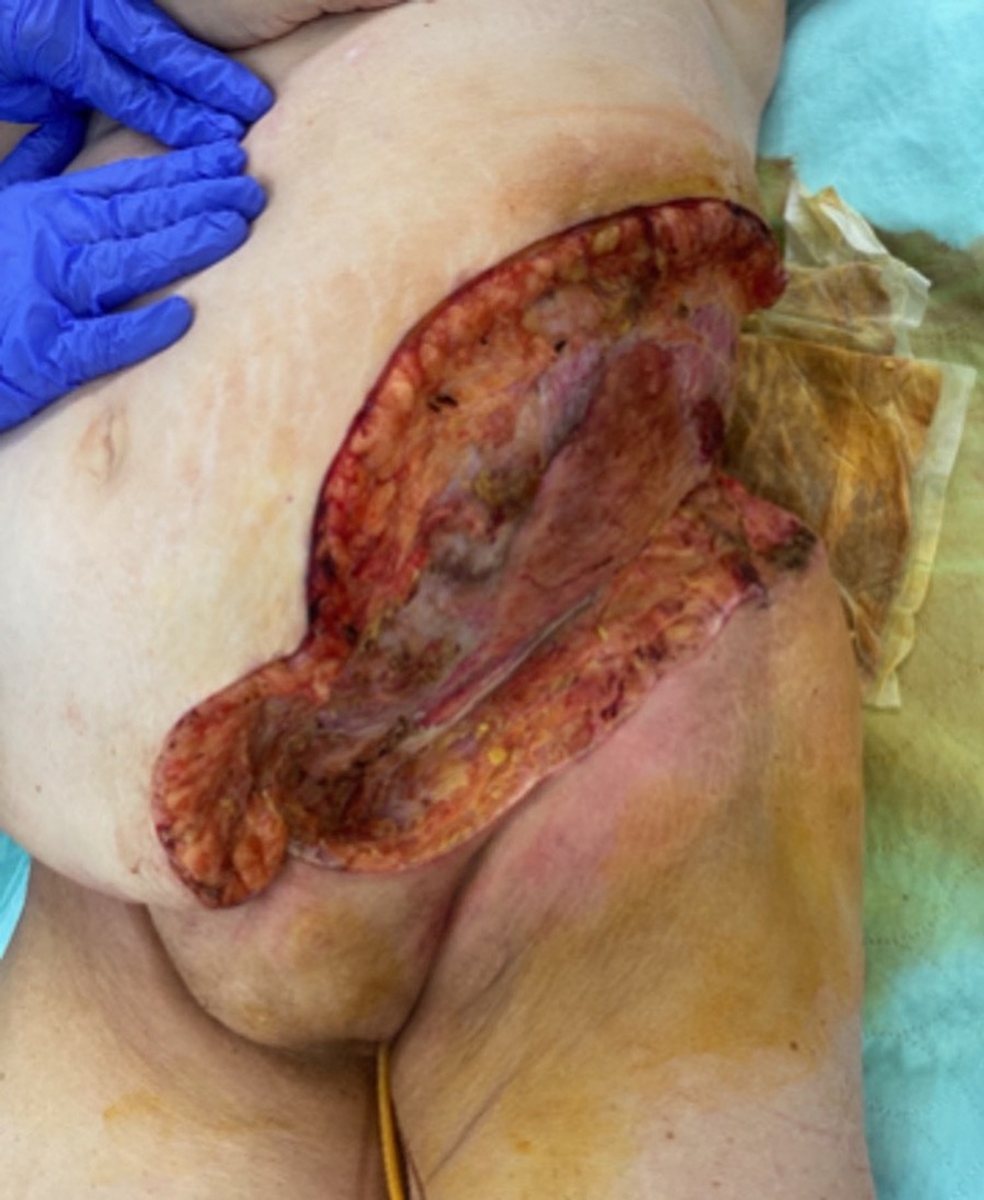
Post-operative day #1 after initial debridement

As a result of these findings, the patient was immediately taken to the operating room for management of a necrotizing soft tissue infection of the left hemi abdomen and left flank. Debridement of skin, soft tissue, fascia, and muscle was performed as shown in Figure 3. 

**Figure 4:**
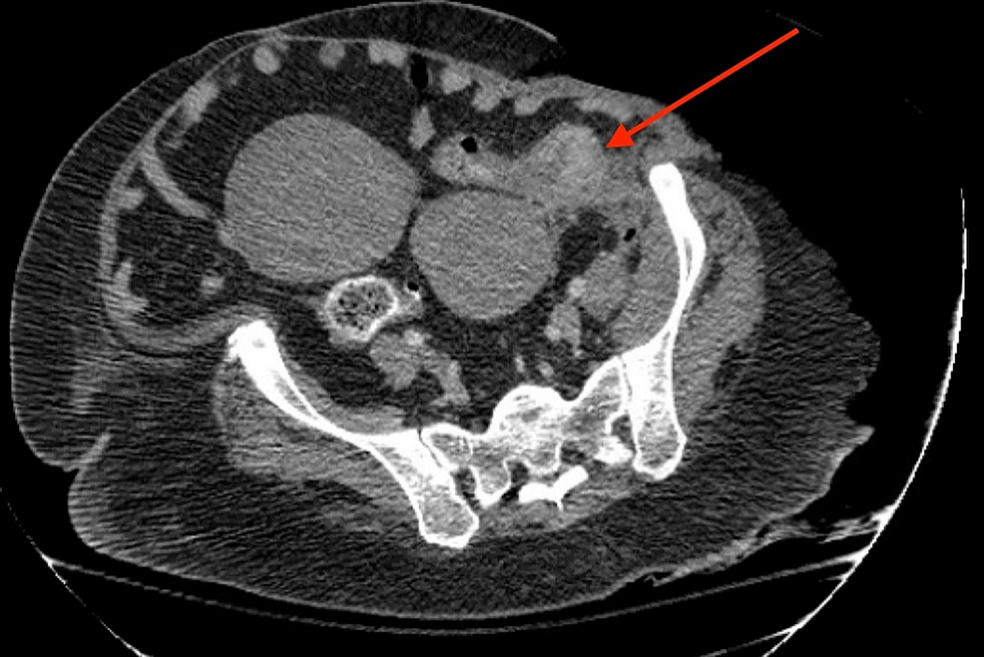
CT abdomen and pelvis showing thickening of sigmoid colon

After further review of the initial CT on the following day, there was suspicion that there was a perforation secondary to a colonic malignancy as evidenced by soft tissue thickening along the wall of the sigmoid colon. Thus, a CT of the abdomen and pelvis with rectal contrast was ordered which showed intraabdominal abscesses and concerns for a tumor within the sigmoid to the descending colon, as seen in Figure 4. Further debridement was continued with the drainage of the intraabdominal abscesses along with a takedown of the splenic flexure, and an open left hemicolectomy with the creation of an end colostomy. During the operation, a perforation of the colon was identified. 

Two days later, a final debridement was completed with the placement of a wound VAC.

**Figure 5:**
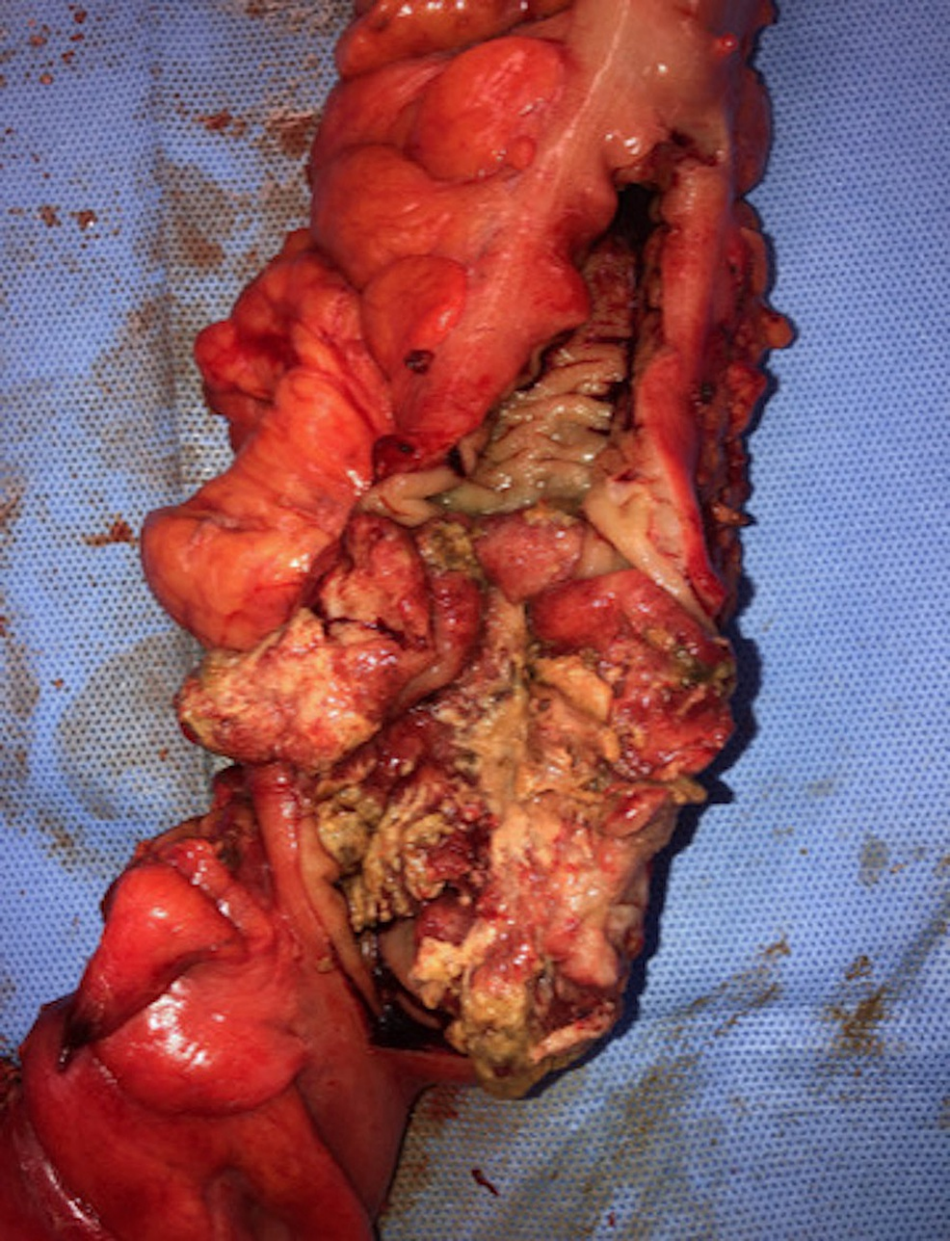
Sigmoid colon cancer specimen

Blood and specimen cultures grew *Streptococcus anginosus* (*S. anginosus*)and final pathology of the specimen demonstrated well-differentiated colonic adenocarcinoma measuring 6.5 cm in length and 1.1 cm in thickness, invading into the muscularis propria but not into the pericolonic fat or serosa. All fourteen lymph nodes were negative for malignancy, stage pT2N0M0 with all resection margins free of tumor. The surgical specimen is shown in Figure 5. 

**Figure 6:**
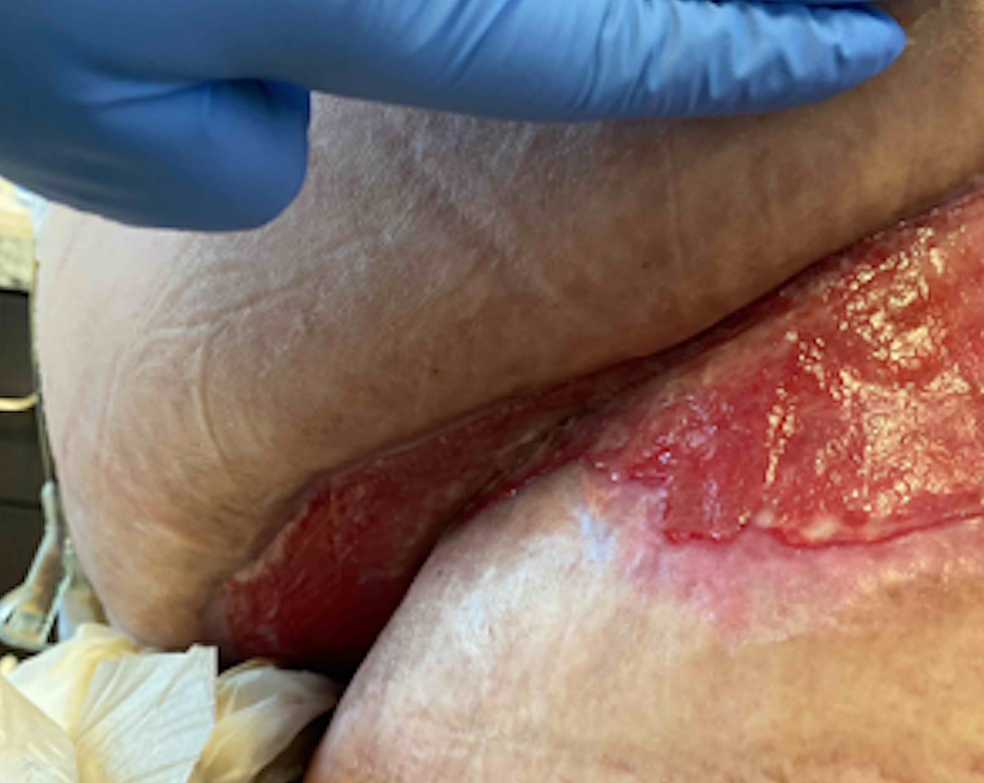
Wound healing at 2.5 months

Post-operatively, the patient was managed with broad-spectrum antibiotics and diabetes management with subcutaneous insulin. She was also followed up for surgical wound care and continuous antibiotics at a long-term acute care facility for two months. Follow-up wound healing at 2.5 months can be seen below in Figure 6.

## Discussion

During the work-up of necrotizing soft tissue infections, differential diagnoses should be considered and ruled out before making a diagnosis of a necrotizing soft tissue infection associated with *S. anginosus* bacteremia, as they have different treatment methods.

One common differential is gas gangrene or NF caused by *Clostridium* species [8]. This type of NF involves both anaerobes and aerobes, an important distinction because this will almost always result in amputation, in contrast to solely debriding, where salvaging the tissue may be possible [8]. 

Our case report is part of a unique subset of cases demonstrating the relationship between the *S. anginosus* species, colon cancer, and NF [9]. There was suspicion that a cancerous lesion disrupted the colonic mucosa, therefore, allowing pathogens to invade the tissue, leading to the conclusion that the infection was a consequence of cancer [9]. In one study, a patient was found to have *S. anginosus* bacteremia with liver abscesses and was incidentally found to have rectal adenocarcinoma, suggesting a similar association [10]. A literature review by Lin et al. revealed only a few case reports showing this relationship [9,10]. In addition, systemic *S. anginosus* infections have also been reported in patients with esophageal and gastric cancer [9,11]. 

When considering treatment options for a necrotizing soft tissue infection, it is important to note that administering antibiotic treatment without debridement is associated with a mortality rate approaching 100% [9]. A major challenge and consequence of lack of prompt diagnosis is delaying the surgical treatment, which correlates with worse outcomes and higher mortality rates [12]. Therefore, surgical source control should be achieved promptly [12]. The mainstay treatment of NSTI is early and aggressive surgery and debridement of necrotic tissue, along with broad-spectrum empiric antibiotic therapy and hemodynamic support [13]. In one review, the only variables that correlated with increased mortality were age and debridement that occurred 24 hours after admission [12,13].

## Conclusions

This study demonstrates proper surgical management for a unique case of NF, cellulitis, and myositis complicated by undiagnosed diabetes mellitus and with an underlying colonic malignancy. While prompt debridement is essential for NSTI, it is important to keep in mind any potential underlying pathologies. Thoroughly reviewing all imaging and searching for other possible etiologies is also necessary for providing the most optimal outcome. Previous studies have shown similar management of NF cases but not with the complications of diabetes mellitus or malignancy. Further studies should explore surgical debridement and long-term follow-up in these specific patient populations with larger sample sizes to help develop best practices for approaching these types of cases.
